# Mutational Profile of *GNAQ* ^Q209^ in Human Tumors

**DOI:** 10.1371/journal.pone.0006833

**Published:** 2009-08-31

**Authors:** Simona Lamba, Lara Felicioni, Fiamma Buttitta, Fonnet E. Bleeker, Sara Malatesta, Vincenzo Corbo, Aldo Scarpa, Monica Rodolfo, Margaret Knowles, Milo Frattini, Antonio Marchetti, Alberto Bardelli

**Affiliations:** 1 Laboratory of Molecular Genetics, Institute for Cancer Research and Treatment, University of Torino Medical School, University of Torino, Medical School Candiolo (TO), Torino, Italy; 2 Clinical Research Center, Center of Excellence on Aging, University-Foundation, Chieti, Italy; 3 Neurosurgical Center Amsterdam, Academic Medical Center (AMC), University of Amsterdam, Amsterdam, The Netherlands; 4 Department of Pathology, Section of Anatomic Pathology, University of Verona, Verona, Italy; 5 Department of Experimental Oncology, Istituto Nazionale Tumori, Milan, Italy; 6 Section of Experimental Oncology, Leeds Institute for Molecular Medicine, Leeds, United Kingdom; 7 Laboratory of Molecular Diagnostic Institute of Pathology, Locarno, Switzerland; 8 Fondazione Italiana per la Ricerca sul Cancro (FIRC) Institute of Molecular Oncology, Milan, Italy; Deutsches Krebsforschungszentrum, Germany

## Abstract

**Background:**

Frequent somatic mutations have recently been identified in the ras-like domain of the heterotrimeric G protein α-subunit (*GNAQ*) in blue naevi 83%, malignant blue naevi (50%) and ocular melanoma of the uvea (46%). The mutations exclusively affect codon 209 and result in *GNAQ* constitutive activation which, in turn, acts as a dominant oncogene.

**Methodology:**

To assess if the mutations are present in other tumor types we performed a systematic mutational profile of the *GNAQ* exon 5 in a panel of 922 neoplasms, including glioblastoma, gastrointestinal stromal tumors (GIST), acute myeloid leukemia (AML), blue naevi, skin melanoma, bladder, breast, colorectal, lung, ovarian, pancreas, and thyroid carcinomas.

**Principal Findings:**

We detected the previously reported mutations in 6/13 (46%) blue naevi. Changes affecting Q209 were not found in any of the other tumors. Our data indicate that the occurrence of *GNAQ* mutations display a unique pattern being present in a subset of melanocytic tumors but not in malignancies of glial, epithelial and stromal origin analyzed in this study.

## Introduction

Activation of the MAPK signaling pathway plays an important role in tumorigenesis. Multiple components of this pathway such as *H, N, K-RAS* and *BRAF* are often mutated in human cancer [1].

Most melanocytic neoplasms show oncogenic mutations in components of the MAPKinase cascade, particularly in *BRAF* and *NRAS* [2]. A recent study has reported frequent somatic mutations in the heterotrimeric G protein α-subunit (*GNAQ*) in a subset of melanocytic neoplasms which do not present alterations in the *RAS* or *BRAF* genes [3]. Genetic, biochemical and biological analysis has shown that *GNAQ* behaves as a bona fide human oncogene. The reported mutations occur exclusively in codon 209 in the ras-like domain and lead to constitutive activation [3]. The glutamine at codon 209 of *GNAQ* corresponding to residue 61 of *RAS* and is essential for GTP hydrolysis [3]. It has been previously shown that in other *RAS* family members, mutations at this site cause loss of GTPase activity with constitutive activation [3].

*GNAQ* encodes for alpha subunit of q class of heterotrimeric GTP binding protein (Gq) that mediates signals between G-protein-coupled receptors (GPCRs) and stimulates all four isoforms of β phospholipase C (PLCβ) that catalyzes the hydrolysis of phosphatidylinositol biphosphate (PIP2). Nearly 40% of GPCRs rely upon Gqα family members to stimulate inositol lipid signalling. These include more then 50 subtypes of receptor responsive to a range of hormone, neurotransmitters, neuropeptides, chemokines, autocrine and paracrine molecules [4]. The Gq family members, Gq, G11, G14, and G15/16, like all heterotrimeric G proteins, are composed of three subunits, Gα, Gβ and Gγ, that cycle between inactive and active signalling states in response to guanine nucleotides. *Gqα (GNAQ), G11α (GNA11), G14*α *(GNA14)* and *G15*α *(GNA15)* each have very different tissue and cell expression patterns. Gqα and G11α mRNA and protein are ubiquitously distributed across tissues [4]. Compared with Gqα human, G11α, G14α, and G15α share 90%, 80%, and 57% amino acid sequence identity, respectively (Table 1). While *GNAQ* and *GNA11* are ubiquitously expressed, other members of the family show a very restricted pattern of expression. For example *GNA 15* is confined to tissues rich in cell types of hematopoietic origin and are enriched in cells in the earlier stages of differentiation [5]. *GNA 14* has been demonstrated to be expressed mainly in kidney, liver, lung and pancreas [4]. Of note exon 5 of *GNA11* contains an equivalent residue to Q209 of *GNAQ*. We hypothesized that mutations in *GNAQ* may also be present in tumor types from non melanocytic origin where they could represent alternative route to MAPKinase activation. To assess this hypothesis, we performed a systematic mutational profile of exon 5 of the *GNAQ* gene in a large panel of human tumors from different tissue types (Table 2). In light of its ubiquitous expression we also performed the analysis of the *GNA11* gene (exon 5) in the same tumor set.

**Table 1:** Sequence homology (at the protein level) and expression distribution in human tissues among Gq family genes.

Gqα family member	*Gqα*	*G11α*	*G14α*	*G15α*
Tissue distribution	Ubiquitous	ubiquitous	kidney, liver, lung, pancreas	Hematopoietic cells
Sequence homology among different Gqα family members	100%	90%	80%	57%

**Table 2:** Number of samples analyzed for each histological type and the number of mutations identified.

Tumor type	Histotype	Number of samples analysed	Number of *GNAQ* mutated samples	P-value Fisher's exact test)
**Blue neavi**		**13**	**6**	
**AML**		**80**	**0**	**2,25E-06**
**Bladder**	**Total**	**39**	**0**	**8,42E-05**
	transitional cell carcinoma	35	0	
	cell line	4	0	
**Breast**	**Total**	**148**	**0**	**7,79E-08**
	ductal carcinoma	61	0	
	lobular carcinoma	50	0	
	medullary carcinoma	18	0	
	mucinous carcinoma	19	0	
**Colorectal**	adenocarcinoma	**119**	**0**	**2,62E-07**
**GIST**		**22**	**0**	**1,06E-03**
**Glioma**	**Total**	**131**	**0**	**1,54E-07**
	glioblastoma	117	0	
	anaplastic astrocytoma	2	0	
	anaplastic oligodendroglioma	2	0	
	high grade glioma cell lines	14	0	
**Lung**	**Total**	**134**	**0**	**1,36E-07**
	adenocarcinoma	110	0	
	small cell carcinoma	17	0	
	carcinoid	7	0	
**Melanoma**	**Total**	**24**	**0**	**7,38E-04**
	primary	1	0	
	nodal metastasis	14	0	
	cutaneous metastasis	7	0	
	visceral metastasis	2	0	
**Ovary**	serous adenocarcinoma	**51**	**0**	**2,28E-05**
**Pancreas**	ductal adenocarcinoma	**98**	**0**	**7,57E-07**
**Thyroid**	**Total**	**63**	**0**	**7,85E-06**
	medullary carcinoma	23	0	
	papillary carcinoma	20	0	
	follicular carcinoma	20	0	

**(AML**: acute myeloid leukemia, **GIST**: Gastrointestinal Stromal Tumors; In addition, p-values of the Fisher's exact test, used to determine the tissue specificity for *GNAQ* ^Q209^ mutations in blue naevi, are listed.)

## Materials and Methods

### Tumor sample and Ethics Statement

DNA of blue naevi, breast, lung, ovarian and thyroid (papillary and follicular isotopes carcinoma) cancer samples was obtained from the Clinical Research Center, Center of Excellence on Aging at the University-Foundation (Chieti, Italy). Samples were collected according to the ethical requirements and regulations of the review board of the Clinical Research Center, University-Foundation (Chieti, Italy).

DNA of melanoma, colorectal cancer and GIST samples was obtained from the Department of Experimental Oncology at the Institute National Tumori (Milan, Italy). Samples were collected according to the ethical requirements and regulations of the review board of the Istituto Nationale dei Tumori (Milan, Italy). DNA of pancreatic adenocarcinoma (PDAC) was obtained from the Department of Pathology, Section of Anatomic Pathology at the University of Verona (Verona, Italy). Samples were collected according to the ethical requirements and regulations of the review board of the University of Verona (Verona, Italy). Additional DNA samples of thyroid carcinomas (medullary histotype), were obtained from the Department of Cellular Biology and Molecular Pathology at the University of Naples (Naples, Italy). Samples were collected according to the ethical requirements and regulations of the review board of the University of Naples (Naples, Italy). DNA of bladder cancer samples was obtained from the Section of Experimental Oncology at the Leeds Institute for Molecular Medicine (Leeds, United Kingdom). Samples were collected according to the ethical requirements and regulations of the review board Institute for Molecular Medicine (Leeds, United Kingdom). Brain cancer samples were obtained from patients undergoing brain tumor surgery in the Academic Medical Center (Amsterdam, The Netherlands). Consent for removal of the tissue and its storage in the tumor bank for research purposes was obtained and documented in the patient's medical chart. Individual consent for this specific project was waivered by the Academic Medical Center (Amsterdam, The Netherlands) ethics committee because the research was performed on ‘waste’ material, stored in a coded fashion. The entire tumor database is described in Table 2.

### Isolation of Genomic DNA and mutational analysis

Genomic DNA was isolated as previously described [6]. PCR primers were designed using Primer 3 (http://frodo.wi.mit.edu/cgi-bin/primer3/primer3_www.cgi), and synthesized by Invitrogen/Life Technologies, Inc. (Paisley, England). A universal sequencing primer M13 forward, (5′-GTAAAACGACGGCCAGT-3′) was appended to the 5′ end used to sequencing (table 3). PCR products size ranged from 180 to 280 bps.

**Table 3:** PCR primers used for the mutational profiling.

Gene	Exon	Forward Primer Sequence	Reverse Primer Sequence
GNAQ	5	5′- TTAATATGAGTATTGTTAACCTTGCAG -3′	5′- M13_CCATTGCCTGTCTAAAGAACAC -3′
GNA11	5	5′- M13_GCCAGGTGGCTGAGTCCT -3′	5′- ACTGCACACAGCCCAAGG-3′

PCRs were performed in 10-uL reaction volumes in 96-well format containing 0.25 mmol/L deoxynucleotide triphosphates, 1 umol/L each of the forward and reverse primers, 6% DMSO, 1×PCR buffer, 1 ng/uL DNA, and 0.05 unit/uL AmpliTaq Gold DNA polymerase (Applied Byosystems, Foster City, CA) A touchdown PCR program was used for PCR amplification (Peltier Thermocycler, PTC-200, MJ Research, Bio-Rad Laboratories, Inc., Italy). PCR products were purified using AMPure (Agencourt Bioscience Corp., Beckman Coulter S.p.A, Milan, Italy). Cycle sequencing was carried out using BigDye Terminator v3.1 Cycle Sequencing kit (Applied Biosystems, Foster City, CA) with an initial denaturation at 97°C for 3 min, followed by 28 cycles of 97°C for 10 s, 50°C for 20 s, and 60°C for 2 min. Sequencing products were purified using CleanSeq (Agencourt Bioscience, Beckman Coulter) and analyzed on a 3730 DNA Analyzer, ABI capillary electrophoresis system (Applied Biosystems). Sequence traces were analyzed using the Mutation Surveyor software package (SoftGenetics, State College, PA). Only amplicons meeting quality criteria were analyzed: tumor samples had Phred quality scores of ≥20.

To assess whether these results were statistically significant we performed the Fisher's exact test to determine the tissue specificity for *GNAQ* mutation in blue naevi tumors as compared to the other tumor types.

## Results and Discussion

**Figure 1:**
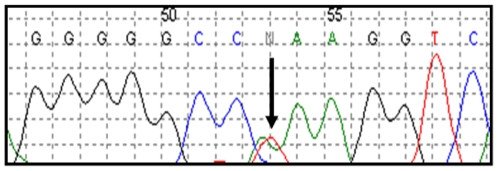
Example of one of the *GNAQ* mutations identified in blue naevi. The arrow indicates the location of the missense change p.Q209L (c.A627T).

We sequenced exon 5 of the *GNAQ* and *GNA11* genes in in a panel of 922 tumors, including glioblastoma, gastrointestinal stromal tumors, acute myeloid leukemia, blue naevi, melanoma, bladder, breast, colorectal, lung, ovarian, pancreas, and thyroid carcinomas (Table 2). The samples included in the analysis have been previously used for mutational profiling of cancer genes and we have shown that common mutations can be identified in this tumors database [6], [7], [8]. A total of 1844 PCR products, spanning 423 kb of tumor genomic DNA, were generated and subjected to direct sequencing. Sequences analysis identified the presence of the Q209L (c.A627T) mutation in *GNAQ* in 6/13 (46%) of blue naevi tumors (Figure 1 and Table 2), thus confirming previous data [3]. Importantly, no mutations of *GNAQ* exon 5 were found in any tumor types, other than blue naevi (Table 2). Similarly, we did not detect mutations in exon 5 of *GNA11*.

To assess whether these results were statistically significant we performed the Fisher's exact test to determine the tissue specificity for *GNAQ* mutation in blue naevi tumor as compared to the other tumor types (Table 2).

Our data confirm that *GNAQ* is a pivotal cancer gene in blue naevi while at the same time unveils a striking tissue-specific pattern of the *GNAQ^Q209^* mutations in human cancer.

Blue naevi arise from intradermal melanocytic proliferations, which can be congenital or acquired, and present in diverse ways ranging from discrete bluish moles (blue naevi) to large blue–grey patches affecting the conjunctiva and periorbital skin (naevus of Ota), shoulders (naevus of Ito) and the lower back (Mongolian spot) [3]. Uveal melanomas are thought to originate from melanocytes within the choroidal plexus of the eye and are distinct from cutaneous melanoma by characteristic cytogenetic alterations. Of note, a potential connection between intradermal melanocytic neoplasms and uveal melanoma is suggested by the fact that naevus of Ota is a risk factor for uveal melanoma [3]. Oncogenic alterations in the *RAS* and *BRAF* genes are known to frequently affect cutaneous melanoma. Until the discovery of the *GNAQ^Q209^* mutations there were no oncogenes altered at high frequency in uveal melanomas and blue naevi. The tissue-specificity of *GNAQ* mutations may be linked to its involvement in endothelin signaling, which is important for development of melanocytes and also is required for the migration of melanoblasts [5]. The canonical downstream signaling pathways of *GNAQ* are the β-isoforms of phospholipase C (PLC-β). Gqα, G11α, bind and stimulate PLC-β enzymes to initiate inositol lipid signalling. PLC-β enzymes catalyze the hydrolysis of the phospholipid phosphatidylinositol bisphosphate, (PIP_2_), to release inositol trisphosphate (IP_3_) and diacylglycerol (DAG). These second messengers propagate and amplify the Gα-mediated signal trough stimulation of protein kinase C (PKC). [3] One hypothesis is that *GNAQ* regulates cell growth through a *RAS* dependent or *RAS*- independent signaling mechanism involving the *PKC*-dependent ERK pathway. [9]. Functional assays are now required to assess this possibility and to understand in details the oncogenic signaling mechanisms regulated by *GNAQ* mutant alleles. Targeting either the mutated *GNAQ^Q209^* protein or the oncogenic signaling pathway controlled by mutated *GNAQ* may open up new therapeutic strategies for melanocytic tumors.

## References

[1] Dunn KatherineL, Espino PaulaS, Drobic Bojan, He Shihua, Davie JamesR (2005). The Ras-MAPK signal transduction pathway, cancer and chromatin remodelling.. Biochem Cell Biol.

[2] Saldanha Gerald, Purnell David, Fletcher Alan, Potter Linda, Gillies Angela (2004). High BRAF mutation frequency does not characterize all melanocytic tumor types.. International Journal of Cancer.

[3] Van Raamsdonk CatherineD, Bezrookove Vladimir, Green Gary, Bauer Jürgen, Gaugleret Lona (2009). Frequent somatic mutations of GNAQ in uveal melanoma and blue naevi.. Nature.

[4] Hubbard KatherineB, Hepler JohnR (2006). Cell signalling diversity of the Gqa family of heterotrimeric G proteins.. Cellular Signalling.

[5] Shin MyungK, Levorse John M, RobertIngram S, ShirleyTilghman M (1999). The temporal requirement for endothelin receptor-B signalling during neural crest development.. Nature Developmental Biology.

[6] Balakrishnan A, Bleeker FE, Lamba S, Rodolfo M, Daniotti M (2007). Novel somatic and germline mutations in cancer candidate genes in glioblastoma, melanoma, and pancreatic carcinoma.. Cancer research.

[7] Bleeker FE, Felicioni L, Buttitta F, Lamba S, Cardone L (2008). AKT1(E17K) in human solid tumours.. Oncogene (2008).

[8] Bleeker FE, Lamba S, Leenstra S, Troost D, Hulsebos T (2009). IDH1 Mutations at Residue p.R132 (IDH1R132) OccurFrequently in High-Grade Gliomas But Not in Other Solid Tumors.. Human Mutation.

[9] Radhika V, Dhanasekaran N (2001). Transforming G proteins.. Oncogene.

